# Expert Delphi survey on research and development into drugs for neglected diseases

**DOI:** 10.1186/1472-6963-11-312

**Published:** 2011-11-16

**Authors:** Angela Fehr, Petra Thürmann, Oliver Razum

**Affiliations:** 1 Dept. of Epidemiology & International Public Health, School of Public Health, Bielefeld University, P.O. Box 10 01 31, 33501 Bielefeld, Germany; 2 University of Witten/Herdecke, Philipp Klee-Institute for Clinical Pharmacology, HELIOS Klinikum Wuppertal, Wuppertal, Germany

## Abstract

**Background:**

Tropical infectious diseases are called neglected, because they are, inter alia, characterized by an R&D deficit. A similar deficit exists for rare (orphan) diseases which neither promise a sufficient return on R&D investment. To encourage the development of treatments for rare diseases, orphan drug acts were created which contain financial and non-financial incentives for the pharmaceutical industry. Similar instruments aimed exclusively at neglected diseases do not yet exist. Proposals for a regulatory approach to promote R&D for neglected diseases include the application of selected orphan drug incentives, or the implementation of a Medical Research and Development Treaty (MRDT) with national funding obligations for medical R&D. We compiled and analyzed experts' opinions on causes for the treatment deficit for neglected diseases and on desirable and feasible measures to promote neglected disease R&D. Hereby, the focus was on mechanisms contained in orphan drug regulations and in the Medical Research and Development Treaty draft (Discussion draft 4, 2005). Lastly, we solicited experts' opinions on the desirability and feasibility of a regulatory instrument to foster R&D for neglected diseases.

**Methods:**

An international online-Delphi survey was conducted with 117 (first round) and 56 (second round) experts of different professional backgrounds and professional affiliations who formulated and ranked causes and solutions related to the treatment deficit for neglected diseases.

**Results:**

In both rounds of survey, the majority of the participating experts (88.4% first round, 86.8% second round) advocated the development of a regulatory instrument to promote R&D for neglected diseases. Most experts (77.9% first round, 79.3% second round) also considered this to be a feasible option. With the exception of market exclusivity, which was viewed critically, key provisions contained in orphan drug regulations were judged favorably also for neglected diseases. A majority (87.1% first round, 77.2% second round) supported national funding obligations for neglected diseases which are proposed by the Medical Research and Development Treaty draft.

**Conclusions:**

While not all features of orphan drug regulations and of the MRDT draft received equal support, the view was expressed that a regulatory instrument would be a desirable and feasible measure to promote R&D for neglected diseases.

## Background

About one billion people worldwide suffer from so-called neglected diseases (NDs). Neglected diseases are a heterogeneous [1] group of predominantly, but not exclusively, tropical infectious diseases, such as Buruli ulcer, Blinding Trachoma, Dengue and Dengue Hemorrhagic Fever, Lymphatic Filariasis, Human African Trypanosomiasis, Onchocerciasis, or Schistosomiasis (a list of 15 NDs is available on http://www.who.int/neglected_diseases/diseases/en/). Common features of neglected diseases include their prevalence in poor populations in developing countries, their cause for stigma and discrimination, their high impact on morbidity and mortality and their relative neglect in terms of research and development activities. For some neglected diseases, tools are available to control, prevent or possibly eliminate them. For other so-called tool-deficient diseases, such tools are still lacking [2,3]. With their often chronic and crippling character, neglected diseases cause immense suffering for the individual patient. Their accumulated prevalence puts a severe strain on the affected societies, with serious and long-lasting social and economic adverse effects on the endemic regions [3]. Substandard living conditions, lack of clean water, sanitary facilities and access to health care contribute to their spread and persistence. Poverty, equivalent to the absence of purchasing power, is considered the main cause for the structural deficit that exists for drug R&D into neglected diseases [2,4-12]. The World Health Organization (WHO) therefore not only speaks of neglected diseases, but also of "neglected populations" [13]. Owing to the lack of a market perspective for pharmaceutical products, R&D efforts for neglected diseases are not proportionate to the diseases' prevalence and their public health impact [14,15]. A similar R&D deficit exists for rare diseases whose small patient populations neither promise adequate returns on investment [16-19]. Public health policy responded to the treatment deficit for rare diseases with the adoption of orphan drug acts, i.e. regulatory instruments with financial and non-financial incentives for the pharmaceutical industry. Comparable legislation focusing on neglected diseases does not yet exist, even though tropical infectious diseases had originally been included both in the concept of "drugs of limited commercial value" [20] which formed the basis for the U.S. Orphan Drug Act, and in early drafts for the European legislation [21]. The first Orphan Drug Act (ODA) was adopted in 1983 in the United States [22], followed by similar legislation in Australia, Japan and, in the year 2000, in the European Union [23]. One incentive of orphan drug legislation is several years of market exclusivity for orphan products. Additionally, sponsors of designated orphan products benefit from fee waivers, protocol assistance and, under the U.S. Orphan Drug Act, from grants and tax credits.

The perceived commonality of rare and neglected diseases, i.e. structural R&D deficits, has nourished a long-standing debate whether orphan drug incentives may also benefit neglected diseases. Owing to their reliance on market exclusivity, however, along with liberty of pricing, orphan drug acts have often been considered ineffective or inappropriate for neglected diseases [21,24-26].

A different regulatory approach to promote R&D for neglected diseases is included in the Medical Research and Development Treaty draft [27]. This document, which was submitted to the WHO Commission on Intellectual Property, Innovation and Public Health (CIPIH) in February 2005 [28], does not exclusively address R&D for neglected diseases; instead, it aims to restructure funding for medical research and development entirely, whereby diseases of poverty are given particular attention. Proceeding from the notion that the current system of funding for medical R&D, based on patents and high drug prices to recoup investment, is ineffective, expensive and does not respond to public health needs [28], the authors and sponsors of the Treaty propose national funding obligations for medical R&D based on GDP or per capita income [27].

Both orphan drug-style incentives and the proposed Medical Research and Development Treaty continue to occupy a prominent place in the debate on promoting R&D into neglected diseases [26,29]. Therefore, we considered it timely and of relevant public health interest to explore among experts the desirability and feasibility of a regulatory instrument and of a selection of mechanisms and incentives contained therein.

## Methods

The survey was based on the concept of a Policy Delphi. A Policy Delphi aims to solicit as many different views on an issue as possible to provide decision makers with the broad spectrum of aspects that have to be considered in a decision-making process [30]. To this end, it follows the characteristic Delphi process of anonymous rounds of survey with feedbacks after each round. A Policy Delphi usually involves three to five rounds, with extensive analyses of dissensions. Guidelines for its implementation recommend *i.a*. at least two professionals to design/monitor the exercise, sometimes prior development of scenarios, factual summaries of background information, pre-tests, presenting respondents their original vote, as well as ensuring that the panel represents a peer group. As Turoff predicts for Policy Delphis, where "the respondents feel strongly about the issues, and this should be the case, [...]", "[...] the questionnaire for the second round will be five to ten times that of the first round.", acknowledging that the items that were contributed later do not receive the same treatment as those listed from the beginning of the survey [30]. We designed the survey based on these guidelines, yet limited the number of rounds to two, with utmost effort devoted to the analysis of suggestions received between the rounds. The questionnaire for the first round was pretested and the initial invitation to participate in the survey was accompanied by a brief project description. The respondents were not shown their votes from previous rounds, though, as this would have required that their anonymity vis-à-vis the research team is lifted; we decided to maintain anonymity as we had stated.

A literature search and document analysis preceded the design of the questionnaire for the first round of survey. Five individuals of different academic backgrounds pretested the first questionnaire and contributed 21 technical and editorial comments. 530 potential participants were initially identified of whom we attempted to contact 388 experts. The following sources were used to compile the panel of experts:

• Publications on neglected and orphan diseases

• Participants in the Conference on Neglected Infectious Diseases, organized by the DG Research of the European Commission, Brussels, November 8 and 9, 2006, which AF attended http://ec.europa.eu/research/health/infectious-diseases/neglected-diseases/pdf/nid-conference-final-report052007_en.pdf

• Contributions to two online hearings (November 1-15, 2006 and August 15 - September 30, 2007) of the WHO Intergovernmental Working Group (WHO-IGWG) to develop a global strategy and plan of action for neglected diseases http://www.who.int/phi/public_hearings/en/

• A letter signed by 162 scientists, public health experts, lawyers, economists, government representatives and parliamentarians to accompany the submission of the Medical Research and Development Treaty draft to the WHO Commission on Intellectual Property, Innovation and Public Health in 2005.

The survey was conducted online in two rounds (March 8-April 3, 2008 and July 10-August 15, 2008; see Additional File 1 Additional File 2: Questionnaires Round I and Round II) using Globalpark EFS Survey 5.2 [31]. In total, the questionnaires contained seven (six in the second round) closed-ended questions, nine full-text fields for items to be added to the questionnaire, one closed-ended question with three options for reply, two hybrid questions and three (six in the second round) full-text fields for general comments. In a qualitative analysis [32] between the first and the second round, full-text suggestions were structured and grouped into categories from which new items were developed and incorporated into the second-round questionnaire. Feedbacks, a key feature of the Delphi process [33], consisted of graphic illustrations of frequency analyses and of pdf-documents with the contents of the nine full-text fields and the three comment sections; both were integrated into the questionnaire for the second round and could be accessed via links. Following the second and last round of the survey, the experts received a link to the survey software to view frequency distributions and comments of the second round. This final feedback closed the exercise. The panel was anonymous so that the replies could not be attributed to the survey participants. For reasons of anonymity, intraindividual variations in the respondents replies between the first and the second round of survey were not tracked. Statistical analyses were performed using Microsoft Excel and SPSS for tabular and graphic presentation.

## Results

### Participation, attrition, demographic data

Of the 388 experts whom we attempted to contact, 159 experts initially agreed to participate in the first round of the survey; of these, 117 fully completed the first questionnaire, and 56 the first and the second questionnaire. Professional backgrounds of the experts in both rounds were in medicine (36.4%/32.1%), public health (30.5%/26.85%), biology/biomedical sciences (30.4%, only round two), pharmaceutical sciences (17.9%, only round two), law (8.5%/10.7%), political science (7.6%/12.5%), economy (4.2%/0%), veterinary medicine (1.8%, only round two) and 'other' (33.1%/8.9%). Multiple responses were possible; biology/biomedical sciences, pharmaceutical sciences and veterinary medicine were added in the second round based on full-text details given in the "other"-field during the first round.

Data on professional affiliations of the experts for both rounds revealed affiliations with academia (53.6%/54.7%), industry (10.7%/11.3%), international organizations (4.5%/7.5%), national governments/parliaments (5.4%/7.5%), non-governmental organizations (14.3%/11.3%), public private partnerships (3.8%, only round two) and 'other' (11.6%/3.8%). The category 'public private partnership' was added in the second round following full-text entries in the first round. Based on publically available data for the 388 experts we initially attempted to contact, we presumed that 48.2% (n = 187) were from academia, 17.5% (n = 68) from national governments/parliaments), 13.9% from non-governmental organizations, 8.5% (n = 33) from industry, 8.3% (n = 32) from backgrounds we were unable to specify ('other'), as well as 3.6% (n = 14) from international organizations. Of the participants in both rounds, 74.8% (75.5%) indicated their place of residency in a developed country, 18.9% (20.8%) in a developing country, and 6.3% (3.8%) in a threshold country/emerging market.

To benefit from answers by experts who abandoned the questionnaire during the course of the survey, frequency distributions were based on N = 159 for the first and N = 77 for the second round, with valid n calculated for each questionnaire item.

### Causes for the treatment deficit for neglected diseases

The first chapter in both rounds of survey dealt with the importance of likely causes for the treatment deficit for neglected diseases. In the first round, seven causes, retrieved from literature search and document analysis were listed; following the experts' suggestions for additional items, the list was expanded to 15 items in the second round (Table 1).

**Table 1:** Causes for the treatment deficit for neglected diseases: Round I and II

		% (n)	% (n)	% (n)	% (n)	% (n)		
		most important	important	unimportant	least important	no judgment	Total valid % (n)	Total N (Missing)

No or insufficient sustainability of public funding for R&D for neglected diseases	Round I	40,3% (48)	47,9% (57)	7,6% (9)	0,8% (1)	3,4% (4)	100,0% (119)	159 (40)

	*Round II*	*54,1% (33)*	*41,0% (25)*	*3,3% (2)*	*1,6% (1)*	*0,0% (0)*	*100% (61)*	*77 (16)*

No or inadequate direct public funding for research and development (R&D) for neglected diseases	Round I	35,2% (44)	60,0% (75)	3,2% (4)	0,0% (0)	1,6% (2)	100,0% (125)	159 (34)

	*Round II*	*54,8% (34)*	*40,3% (25)*	*4,8% (3)*	*0,0% (0)*	*0,0% (0)*	*100% (62)*	*77 (15)*

No or inadequate incentives for the private sector to invest into R&D for neglected diseases	Round I	33,9% (42)	49,2% (61)	8,9% (11)	4,8% (6)	3,2% (4)	100,0% (124)	159 (35)

	*Round II*	*41,9% (26)*	*45,2% (28)*	*8,1% (5)*	*3,2% (2)*	*1,6% (1)*	*100% (62)*	*77 (15)*

No or inadequate private sector investment into R&D for neglected diseases	Round I	33,6% (42)	58,4% (73)	4,8% (6)	0,8% (1)	2,4% (3)	100,0% (125)	159 (34)

	*Round II*	*45,2% (28)*	*40,3% (25)*	*4,8% (3)*	*8,1% (5)*	*1,6% (1)*	*100% (62)*	*77 (15)*

No or inadequate access to effective drugs for neglected diseases	Round I	33,1% (40)	47,1% (57)	9,1% (11)	8,3% (10)	2,5% (3)	100,0% (121)	159 (38)

	*Round II*	*41,0% (25)*	*47,5% (29)*	*4,9% (3)*	*6,6% (4)*	*0,0% (0)*	*100% (61)*	*77 (16)*

No or inadequate research infrastructure in countries with neglected diseases	Round I	29,5% (36)	55,7% (68)	7,4% (9)	6,6% (8)	0,8% (1)	100,0% (122)	159 (37)

	*Round II*	*27,9% (17)*	*60,7% (37)*	*8,2% (5)*	*3,3% (2)*	*0,0% (0)*	*100% (61)*	*77 (16)*

No or ineffective drugs for neglected diseases	Round I	20,5% (24)	48,7% (57)	15,4% (18)	8,5% (10)	6,8% (8)	100,0% (117)	159 (42)

	*Round II*	*30,0% (18)*	*58,3% (35)*	*8,3% (5)*	*3,3% (2)*	*0,0% (0)*	*100% (60)*	*77 (17)*

Disease-specific research difficulties (unknown etiology, lack of research material)	*Round II*	*4,9% (3)*	*62,3% (38)*	*18,0% (11)*	*11,5% (7)*	*3,3% (2)*	*100% (61)*	*77 (16)*

No or inadequate research coordination	*Round II*	*8,2% (5)*	*49,2% (30)*	*32,8% (20)*	*6,6% (4)*	*3,3% (2)*	*100% (61)*	*77 (16)*

Lack of awareness/visibility of neglected diseases	*Round II*	*32,8% (20)*	*52,5% (32)*	*13,1% (8)*	*1,6% (1)*	*0,0% (0)*	*100% (61)*	*77 (16)*

Lack of health-needs driven priority setting in public funding	*Round II*	*44,3% (27)*	*50,8% (31)*	*4,9% (3)*	*0,0% (0)*	*0,0% (0)*	*100% (61)*	*77 (16)*

No or inadequate health delivery infrastructure and staff in developing countries	*Round II*	*45,9% (28)*	*41,0% (25)*	*9,8% (6)*	*1,6% (1)*	*1,6% (1)*	*100% (61)*	*77 (16)*

Inadequate research priorities in private sector R&D	*Round II*	*48,4% (30)*	*37,1% (23)*	*11,3% (7)*	*3,2% (2)*	*0,0% (0)*	*100% (62)*	*77 (15)*

Poverty as reason for market failure (perception of no market for drugs, insufficient R&D)	*Round II*	*55,0% (33)*	*35,0% (21)*	*5,0% (3)*	*3,3% (2)*	*1,7% (1)*	*100% (60)*	*77 (17)*

Poverty as disease-proliferating factor (i.a. inadequate prevention, inadequate housing, lack of clean water) in endemic countries	*Round II*	*57,4% (35)*	*32,8% (20)*	*6,6% (4)*	*3,3% (2)*	*0,0% (0)*	*100%(61)*	*77 (16)*

The results for those items that were added to the second round of survey are shown in the lower part of the table

The experts were rather agreed on the importance of the causes, whereby in the first round of survey both public and private funding deficits were rated slightly more important than the lack of access to existing drugs, inadequate R&D infrastructure in endemic countries or the absence of effective drugs. In the second round of survey, two newly-added causes, labeled 'poverty as a disease-proliferating factor' and 'poverty as the reason for market failure', were considered the most important causes for the treatment deficit; nearly equal importance was attributed to the lack of (sustainable) public R&D funding, which had been ranked first in the initial round of survey. 13 of the 15 causes listed in the second round were considered most important or important by more than 80% of the panelists; only two items, i.e. disease-specific research difficulties and no or inadequate research coordination received less than 80% of aggregated positive replies.

### Orphan drug regulations for rare diseases

The first question in this section, which asked if and to which extent the respondents were familiar with orphan drug laws, served as a filter question. Experts with no knowledge about these laws (n = 53) skipped the question on their effectiveness, while those with active (n = 13) and with passive knowledge (n = 60) continued to the assessment. 61.4% of the respondents rated orphan drug regulations very effective or effective; of their individual incentives, market exclusivity was given the highest ranking (22.1%) in the category "very effective"; the highest aggregated positive reply (very effective/effective) was given to tax credits (62.3%). (Table 2)

**Table 2:** Effectiveness of orphan drug laws and incentives

	very effective	effective	ineffective	very ineffective	no judgment
Orphan drug laws	7.1%	54.3%	17.1%	0%	21.4%

Market exclusivity	22.1%	33.8%	17.6%	4.4%	22.1%

Tax credits	14.5%	47.8%	10.1%	1.4%	26.1%

Protocol assistance	13.2%	41.2%	14.7%	0%	30.9%

Fee reduction/Fee waivers	8.8%	41.2%	23.5%	1.5%	25.0%

### Measures to promote R&D for neglected diseases

In the next chapter of the questionnaire, the participating experts were requested to rank (according to desirability and feasibility) a list of possible measures to promote R&D for neglected diseases. Three key incentives contained in orphan drug regulations, i.e. tax credits, fee waivers and protocol assistance, or scientific advice, were considered desirable and feasible to foster R&D for neglected diseases by more than two thirds of the experts in both rounds. (Table 3, Table 4)

**Table 3:** Desirability of orphan drug incentives for neglected diseases

		% (n)	% (n)	% (n)	% (n)	% (n)		
		very desirable	desirable	undesirable	very undesirable	no judgment	Total valid % (n)	Total N (Missing)

Market exclusivity	Round I	7,8% (9)	19,1% (22)	26,1% (30)	20,9% (24)	26,1% (30)	100% (115)	159 (44)

	*Round II*	*3,6% (2)*	*20,0% (11)*	*36,4% (20)*	*21,8% (12)*	*18,2% (10)*	*100% (55)*	*77 (22)*

Tax credits	Round I	14,2% (16)	48,7% (55)	6,2% (7)	1,8% (2)	29,2% (33)	100% (113)	159 (46)

	*Round II*	*20,4% (11)*	*46,3% (25)*	*11,1% (6)*	*3,7% (2)*	*18,5% (10)*	*100% (54)*	*77 (23)*

Protocol assistance	Round I	25,9% (30)	44,0% (51)	4,3% (5)	0,0% (0)	25,9% (30)	100% (116)	159 (43)

	*Round II*	*33,9% (19)*	*55,4% (31)*	*3,6% (2)*	*0,0% (0)*	*7,1% (4)*	*100% (56)*	*77 (21)*

Fee reduction/Fee waivers (e.g. for marketing approval, scientific advice)	Round I	27,0% (31)	53,9% (62)	3,5% (4)	0,9% (1)	14,8% (17)	100% (115)	159 (44)

	*Round II*	*28,1% (16)*	*56,1% (32)*	*7,0% (4)*	*0,0% (0)*	*8,8% (5)*	*100% (57)*	*77 (20)*

**Table 4:** Feasibility of orphan drug incentives for neglected diseases

		% (n)	% (n)	% (n)	% (n)	% (n)		
		very (definitely) feasible	feasible	unfeasible	definitely unfeasible	no judgment	Total valid % (n)	Total N (Missing)

Market exclusivity	Round I	10% (11)	28,2% (31)	20,9% (23)	2,7% (3)	38,2% (42)	110	159 (49)

	*Round II*	*13.7% (7)*	*27.5% (14)*	*27.5% (14)*	*2.0% (1)*	*29.4% (15)*	*51*	*77 (26)*

Tax credits	Round I	21.8% (24)	40,0% (44)	4,5% (5)	2,7% (3)	30,9% (34)	110	159 (49)

	*Round II*	*35.3% (18)*	*37.3% (19)*	*5.9% (3)*	*2.0 (1)*	*19.6% (10)*	*51*	*77 (26)*

Protocol assistance	Round I	32.7% (36)	44,5% (49)	1.8% (2)	0,0% (0)	20.9% (23)	110	159 (50)

	*Round II*	*35,3% (18)*	*52.9% (27)*	*3.9% (2)*	*0.0% (0)*	*7.8% (4)*	*51*	*77 (26)*

Fee reduction/Fee waivers (e.g. for marketing approval, scientific advice)	Round I	26,6% (29)	51.4% (56)	4.6% (5)	0,9% (1)	16.5% (18)	110	159 (49)

	*Round II*	*23.1% (12)*	*50.0% (26)*	*11.5% (6)*	*0.0% (0)*	*15.4% (8)*	*52*	*77 (25)*

National funding obligations for medical R&D and prize funds proposed in the Medical Research and Development Treaty draft were supported by a majority of experts in both rounds; the concept of separating innovation incentives from drug prices rendered slightly more controversial results. Perhaps owing to the far-reaching character of these paradigm changes, the survey participants were slightly cautious in predicting their feasibility. (Table 5, Table 6)

**Table 5:** Desirability of MRDT proposals to promote R&D for neglected diseases

		% (n)	% (n)	% (n)	% (n)	% (n)		
		very desirable	desirable	undesirable	very undesirable	no judgment	Total valid % (n)	Total N (Missing)

Prize funds with prizes awarded based on degree of innovation	Round I	35.6% (42)	44.1% (52)	6.8% (8)	3.4% (4)	10.2% (12)	118	159 (41)

	*Round II*	*33.9% (19)*	*35.7% (20)*	*12.5% (7)*	*3.6% (2)*	*14.3% (8)*	*56*	*77 (21)*

Obligation for national governments to invest into neglected disease R&D	Round I	48.3% (56)	38.8% (45)	6.9% (8)	1.7% (2)	4.3% (5)	116	159 (43)

	*Round II*	*40.4% (23)*	*36.8% (21)*	*14.0% (8)*	*1.8% (1)*	*7.0% (4)*	*57*	*77 (20)*

Separation of innovation incentives from drug prices	Round I	38.8% (45)	30.2% (35)	4.3% (5)	4.3% (5)	22.4% (26)	116	159 (43)

	*Round II*	*38.2% (21)*	*27.3% (15)*	*12.7%(7)*	*5.5% (3)*	*16.4% (9)*	*55*	*77 (22)*

**Table 6:** Feasibility of MRDT proposals to promote R&D for neglected diseases

		% (n)	% (n)	% (n)	% (n)	% (n)		
		definitely feasible	feasible	unfeasible	definitely unfeasible	no judgment	Total valid % (n)	Total N

Prize funds with prizes awarded based on degree of innovation	Round I	20.9% (23)	60.0% (23)	4.5% (5)	0.9% (1)	13.6% (15)	110	159

	*Round II*	*30.8% (16)*	*48.1% (25)*	*11.5% (6)*	*1.9% (1)*	*7.7% (4)*	*52*	*77 (25)*

Obligation for national governments to invest into neglected disease R&D	Round I	28.2% (31)	41.8% (46)	20.0% (22)	5.5% (6)	4.5% (5)	110	159 (49)

	*Round II*	*11.3% (6)*	*43.4% (23)*	*35.8% (19)*	*3.8% (2)*	*5.7% (3)*	*53*	*77 (24)*

Separation of innovation incentives from drug prices	Round I	12.6% (14)	39.6% (44)	11.7% (13)	6.3% (7)	29.7% (33)	111	159 (48)

	*Round II*	*15.1% (8)*	*41.5% (22)*	*11.3% (6)*	*9.4% (5)*	*22.6% (12)*	*53*	*77 (24)*

In the first round of survey, we received 134 proposals for modifications of previously listed measures or for additional measures. These covered funding priorities, research cooperation, capacity building and knowledge transfer, access, visibility and awareness, incentives, and structural reforms and regulations. After careful analysis, the proposals were developed into a list of 50 measures for the second round of survey.

### A regulatory instrument to promote R&D for neglected diseases

Further to the questions on causes and measures, it was of interest to learn whether the panelists considered it desirable and feasible to have a regulatory instrument to promote R&D for neglected diseases in which these measures might be absorbed. In both rounds of survey, the respondents found the option (very) desirable as well as feasible. (Table 7)

**Table 7:** A regulatory instrument to promote R&D for neglected diseases: Round I and II

	very desirable	desirable	undesirable	very undesirable	no judgment	n =	missing**	Median***
Desirability*	44.2% (49.1%)	44.2% (37.7%)	7.1% (3.8%)	1.8% (3.8%)	2.7% (5.7%)	113 (53)	46 (24)	2.00 (1.00)

	very (definitely) feasible	feasible	unfeasible	definitely unfeasible	no judgment	n	missing**	Median***

Feasibility*	14.2% (18.9%)	63.7% (60.4%)	8.0% (15.1%)	3.5% (5.7%)	10.6% (0.0%)	113 (53)	46 (24)	2.00 (2.00)

Results of the second round of the survey are shown in brackets

*1 = very desirable/very (definitely) feasible, 2 = desirable/feasible, 3 = undesirable/unfeasible, 4 = very undesirable/definitely unfeasible, 5 = no judgment

**Participants who abandoned the questionnaire prior to this question or who did not answer the question

*** Excludes 5 = no judgment

Cross-tabulations by professional affiliation on the basis of aggregated positive replies (very desirable/desirable) revealed that, in the first round of survey, most subgroups were supportive of the concept of a regulatory instrument (90% academia, 100% national government/parliament, 100% international organizations, 93.8% non-governmental organizations and 100% "other") Of the experts affiliated with industry, 58.3% expressed a positive opinion on a regulatory instrument, while 41.6% said it was undesirable or very undesirable. In the second round of survey, experts with affiliations in academia, national government/parliament, non-governmental organization and other affiliations again gave over 90% of support for a regulatory instrument. Opinions in the subgroup "industry" remained diverse (66.7% for and 33.3% against a regulatory instrument). Also, representatives of international organizations were less agreed in the second round; one representative considered it desirable, one undesirable and two expressed no judgment. Similarly, the two experts affiliated with a public private partnership were disagreed on the subject.

In both rounds of survey, the feasibility of a regulatory instrument was judged positively by experts from academia (80%/85.7%), national governments/parliaments (83.3%/100%) and from NGOs (87.6%/83.3%) In contrast, while 80% of the respondents affiliated with an international organization had considered a regulatory instrument to be a feasible option in the first round, three out of four experts in this subgroup said it was possibly unfeasible in the second round. Experts affiliated with industry were equally skeptical in their assessment of the instrument's feasibility (50%/66.7%).

## Discussion

### Method

The Delphi method and its online implementation were well received. A large number of experts participated in the survey over a period of five months. They devoted time and effort to fill out two online questionnaires and contributed new items as well as extensive - also critical! - comments. The latter ranged from "not sure what you can deduce from this listing - most listed items are clearly important, and there's much overlap, so what are we going to learn from "important" versus "very important"?" to "The results will [be] interesting and helpful for policy development" or: "Very interesting and useful. I guess sometimes one would like more nuanced options for a more appropriate answer".

As we have shown in the first paragraph of the Results section, the sample was not balanced in terms of professional backgrounds, professional affiliations or the place of residence. The aim of the survey was to gather as broad a spectrum of perspectives as possible. The sample selection was purposive insofar as we chose specific sources from which to gather a panel of interested experts, such as relevant publications, conferences, hearings etc.. Prior to the first round of the survey, we could only assume the distribution of experts in the demographic categories. Since it was impossible to predict participation and attrition rates at the onset of the study, we did not attempt to reduce and adjust the initial panel to represent a balanced sample. We do not dare to anticipate how and if the results of the study may have differed if the panel had been more balanced. However, we observed variations within groups in cross-tabulations which suggest that the assessments made by the experts may not necessarily be linked to professional affiliations.

### Results

The survey facilitated the quantification and correlation of views far beyond what can be reproduced in this paper. Its outcome underlined that insufficient funding for R&D for neglected diseases is one of the most important causes for the treatment deficit. Yet it is by no means the only important cause: there was only one item (no/ineffective drugs for NDs) in the first round, and two items (no/inadequate research coordination and disease-specific research difficulties) in the second round that received less than 80% of aggregated positive responses (most important/important) by all participating experts. Similarly, while respondents from all professional affiliations chose funding issues as most important in the first round, they suggested 98 additional items, which formed the basis for the extension of the list of causes in the second round. Consequently, funding issues became one of several causes that received high priority in the different professional affiliations. This process and its outcome underline the complexity of the issue and the need to uphold multifocal strategies that will increase awareness, reduce poverty, adjust R&D priorities to include NDs, increase and sustain public and private sector funding, and promote health as well as research infrastructure in endemic countries.

Certainly, no single mechanism or regulatory instrument can cover this broad range of challenges. The participants of the survey offered a diversity of comments on the desirability and feasibility of a regulatory instrument, including: "This is a purely national decision unlikely to be easily introduced into legislative bodies, but definitely worth trying. Once a few countries install some such instrument others will follow."; "would best be done under the auspices of the WHO, would cover all aspects from discovery to delivery, with follow up monitoring"; "We have too much regulation already and implementation on a global basis is simply not practicable".

Various modifications to orphan drug laws for the benefit of NDs have been suggested in recent years such as transferable market exclusivity [34,35], or the definition of all NDs as orphan diseases [25]. The survey revealed that incentives such as tax credits, fee waivers and protocol assistance were also considered promising to promote R&D into drugs for neglected diseases. In view of the fact that a majority of the respondents advocated a regulatory instrument to promote R&D for neglected diseases, it may be of interest to further explore whether it would be beneficial to extend selected orphan drug incentives to neglected diseases [36]. Correspondingly, for several years, the concept of a biomedical R&D treaty has been debated at international levels [26,28,29,37-39], and discussed in the scientific community [34,40-46]. One of its key concepts, i.e. national funding obligations for medical R&D, was considered desirable by a majority of the survey participants. The feasibility of this proposal, however, was judged more cautiously.

The respondents were not asked, however, to assess orphan drug acts or the MRDT for neglected diseases as such. This, on the one hand, precludes a comprehensive answer to the question of their desirability and feasibility for ND research and development. On the other hand, the separation of individual measures from specific regulatory instruments was part of the survey's concept to enable us to learn which measures are attractive and practicable in the eyes of the respondents. It may thus be of interest to carry forward, expand and focus the research, perhaps with a similar methodology, to engage experts and stakeholders in determining which measures could contribute to a future instrument to promote R&D for neglected diseases.

## Conclusions

The treatment deficit for neglected diseases can neither be attributed to a single cause, nor can it be remedied by a single measure. Both orphan drug acts and the MRDT are controversial approaches to promoting neglected disease R&D. The ongoing debate on the two concepts points to a strong interest in a regulatory instrument, which we could confirm in our Delphi survey. Such instrument may not rely on the same incentives as orphan drug regulations, or completely restructure medical R&D funding as proposed in the MRDT. Still, if it included mechanisms to provide sufficient and sustainable funding for effective, affordable and field-sensitive diagnostics and treatments, facilitated research cooperation and increased awareness for NDs on health policy agendas, it would express the same political will to guarantee equitable health care for patients with neglected diseases as orphan drug regulations intended for patients with rare diseases.

It is recommended that a Policy Delphi is followed by a working group which may utilize the results to formulate relevant policy recommendations. [30] The data which have been gathered in the survey will be analyzed further so that the valuable input of the respondents will contribute to the ongoing search for mechanisms to promote R&D for neglected diseases.

## Competing interests

The authors declare that they have no competing interests.

## Authors' contributions

AF, PT and OR conceived of the survey, and participated in its design and coordination. AF implemented the survey and performed the statistical analysis. AF, PT and OR drafted, read and approved the manuscript.

## Pre-publication history

The pre-publication history for this paper can be accessed here:

http://www.biomedcentral.com/1472-6963/11/312/prepub

## Supplementary Material

Additional file 1 **Questionnaire Round I**. Questionnaire of the first round of the Delphi survey. Click here for file

Additional file 2 **Questionnaire Round II**. Questionnaire of the second round of the Delphi survey. Click here for file
