# Supplementary material for: Expert Delphi survey on research and development into drugs for neglected diseases
Source: BMC Health Serv Res. 2011 Nov 16;11:312. doi: 10.1186/1472-6963-11-312 (PMC3228726; doi:10.1186/1472-6963-11-312)
Supplement: Additional file 1 — Questionnaire Round I. Questionnaire of the first round of the Delphi survey. [file 1472-6963-11-312-S1.PDF]

[9%]

Welcome and thank you for supporting this research project on neglected and orphan diseases.

This is the first of two rounds of the project's Delphi survey. The survey will take about 10 minutes to complete.

The first round can be accessed until March 29, 2008.

Next

Cancel

[9%]

I. Neglected Diseases

Neglected diseases are disease states where there are inadequate, ineffective or no means to prevent, treat, diagnose or cure them. (WHO/CIPIH)

1. What, in your opinion, are the most important causes for this deficit?

Technical advice: Please use your left mouse button to click the button of your choice. You may change your answer by clicking another button.

	most important	important	unimportant	least important	no judgement
a) No or inadequate direct public funding for research and developement (R&D) for neglected diseases	○	○	○	○	○
b) No or inadequate private sector investment into R&D for neglected diseases	○	○	○	○	○
c) No or inadequate incentives for the private sector to invest into R&D for neglected diseases	○	○	○	○	○
d) No or insufficient sustainability of public funding for R&D for neglected diseases	○	○	○	○	○
e) No or ineffective drugs for neglected diseases	○	○	○	○	○
f) No or inadequate access to effective drugs for neglected diseases	○	○	○	○	○
g) No or inadequate research infrastructure in countries with neglected diseases	○	○	○	○	○

2. Are there items you would like to add to this list?

h) ____________________

i) ____________________

j) ____________________

Next

Cancel

[18%]

II. Orphan diseases

For several years, laws or regulations have existed to foster research and development (R&D) for orphan diseases. These diseases are characterized by a very low prevalence which led to deficits in R&D. Orphan drug laws were developed to provide R&D incentives.

1. Are you familiar with orphan drug laws?

Technical advice: Please use your left mouse button to click the button of your choice. You may change your answer by clicking another button.

- ○ Yes, I have active knowledge of the provisions in these laws (e.g. through application processes for orphan drug status)
- ○ Yes, I have passive knowledge of orphan drug laws (e.g. through publications)
- ○ No, I have no knowledge about the provisions contained in orphan drug laws

Back Next

Cancel

[27%]

2. How effective do you consider orphan drug laws?

Technical advice: Please use your left mouse button to make your choice. You may change your answer by clicking another button.

very effective	effective	ineffective	very ineffective	no judgement
○	○	○	○	○

3. How effective are the individual provisions of orphan drug laws?

Technical advice: Please use your left mouse button to make your choice. You may change your answer by clicking another button.

	very effective	effective	ineffective	very ineffective	no judgement
a) Fee reduction / Fee waivers	○	○	○	○	○
b) Market exclusivity	○	○	○	○	○
c) Protocol assistance	○	○	○	○	○
d) Tax credits	○	○	○	○	○

Back Next

Cancel

[36%]

III. New options for neglected diseases?

1. Following is a list of measures to promote medical research and development.

a) How desirable* is it to implement these measures to foster R&D for neglected diseases?
**Please see definition at the bottom of this page.*

Technical advice: Please use your left mouse button to click the button of your choice. You may change your answer by clicking another button.

	very desirable	desirable	undesirable	very undesirable	no judgement
i) Advance market commitments	○	○	○	○	○
ii) Exemption of drugs from market exclusivity	○	○	○	○	○
iii) Existing patent regulations	○	○	○	○	○
vi) Fee reduction / Fee waivers (e.g. for marketing approval, scientific advice)	○	○	○	○	○
v) Investment obligations into neglected diseases for drug producers/sellers	○	○	○	○	○
vi) Market exclusivity	○	○	○	○	○
vii) Obligations for national governments to invest into neglected disease R&D	○	○	○	○	○
viii) Open source regulations (e.g. for scientific data / compound libraries)	○	○	○	○	○
ix) Patent pools	○	○	○	○	○
x) Philantrophic spending	○	○	○	○	○
xi) Prize funds for drug innovation	○	○	○	○	○
xii) Protocol assistance	○	○	○	○	○
xiii) Public-private partnerships	○	○	○	○	○
xiv) Separation of innovation incentives from drug prices	○	○	○	○	○
xv) Tax credits	○	○	○	○	○
xvi) Tiered/differential pricing	○	○	○	○	○

Definition "Desirability"

Very desirable:

extremely beneficial / will have a positive effect and little to no negative effect

Desirable:
beneficial / will have a positive effect and little to no negative effect

Undesirable:
harmful / will have a negative effect

Very undesirable:
extremely harmful / will have a major negative effect

Back Next

Cancel

[45%]

b) How feasible* is it to implement these measures to foster R&D for neglected diseases?
**Please see definition at the bottom of this page.*

Technical advice: Please use your left mouse button to click the button of your choice. You may change your answer by clicking another button.

	very feasible	feasible	unfeasible	very unfeasible	no judgment
i) Advance market commitments	○	○	○	○	○
ii) Exemption of drugs from market exclusivity	○	○	○	○	○
iii) Existing patent regulations	○	○	○	○	○
iv) Fee reduction / Fee waivers (e.g. for marketing approval, scientific advice)	○	○	○	○	○
v) Investment obligations into neglected diseases for drug producers/sellers	○	○	○	○	○
vi) Market exclusivity	○	○	○	○	○
vii) Obligations for national governments to invest into neglected disease R&D	○	○	○	○	○
viii) Open source regulations (e.g. for scientific data / compound libraries)	○	○	○	○	○
ix) Patent pools	○	○	○	○	○
x) Philantrophic spending	○	○	○	○	○
xi) Protocol assistance	○	○	○	○	○
xii) Prize funds for drug innovation	○	○	○	○	○
xiii) Public-private partnerships	○	○	○	○	○
xiv) Separation of innovation incentives from drug prices	○	○	○	○	○
xv) Tax credits	○	○	○	○	○
xvi) Tiered/differential pricing	○	○	○	○	○

Definition "Feasibility"

Definitely feasible:
no hindrance to implementation / no political roadblocks / acceptable to the public

Possibly feasible:
some indication this is implementable / minor political roadblocks / further consideration or preparation to be given to public reaction

Possible unfeasible:
some indication this is unworkable / severe political resistances / difficult to communicate to the public

Definitely unfeasible:
all indications are negative / politically unworkable / cannot be implemented

Back Next

Cancel

55%

2. What other measures not yet in force could promote R&D for neglected diseases? Please list the three most important measures.

a)

b)

c)

Back Next

Cancel

[64%]

3. Do you consider it desirable to have a regulatory instrument (a law, regulation or treaty) to foster R&D for neglected diseases?

Technical advice: Please use your left mouse button to make your choice. You may change your answer by clicking another button.

very desirable	desirable	undesirable	very undesirable	no judgement
○	○	○	○	○

Would you like to comment on your position on this question?
Please fill in the text field.

4. Do you consider it feasible to implement such a regulatory instrument?

Technical advice: Please use your left mouse button to make your choice. You may change your answer by clicking another button.

very feasible	feasible	unfeasible	very unfeasible	no judgment
○	○	○	○	○

Would you like to comment on your position on this question?

Please fill in the text field.

Back Next

Cancel

[73%]

5. Under orphan drug laws, a disease has to meet specific criteria of prevalence and disease severity (e.g. life-threatening, seriously debilitating, serious and chronic condition) to be classified as are rare, or orphan disease.

Which are the criteria a disease would have to meet to be classified as "neglected disease" under a regulatory instrument?

Please name the three most important criteria.

Please fill in the text fields.

1

2

3

Back Next

Cancel

[82%]

IV. Conclusion

1. Do you have any comments on this Delphi survey or on the questionnaire?

2. You have completed the questionnaire. In conclusion, we would be grateful if you gave us the following demographic information.

a) What is your professional background?

Technical advice: Please use your left mouse button to check boxes.
You may check more than one box. You may change your answer by clicking another button.

- ☐ Economy
- ☐ Law
- ☐ Medicine
- ☐ Political Science
- ☐ Public Health
- ☐ Other

b) What is your current professional affiliation?

Technical advice: Please use your left mouse button to check boxes. You may change your answer by clicking another button.

- ○ Academia
- ○ National government/parliament
- ○ Industry
- ○ International organization
- ○ Non-governmental organization
- ○ Other

c) Your place of residence is in a

Technical advice: Please use your left mouse button to check boxes. You may change you answer by clicking another radio-button.

- ○ Developed country
- ○ Developing country
- ○ Threshold country/emerging market

Back Next

Cancel

100%

Thank you for participating in this Delphi survey!

This was the first of two rounds. We will now evaluate the outcome of this round.

All participants who completed the first round will shortly receive an email containing the link to the questionnaire for the second (and last) round of this survey.

In the second round, you will see the results of this first round as well as comments and suggestions which the participating experts have added to this questionnaire.

Close window
