# Supplementary material for: Expert Delphi survey on research and development into drugs for neglected diseases
Source: BMC Health Serv Res. 2011 Nov 16;11:312. doi: 10.1186/1472-6963-11-312 (PMC3228726; doi:10.1186/1472-6963-11-312)
Supplement: Additional file 2 — Questionnaire Round II. Questionnaire of the second round of the Delphi survey. [file 1472-6963-11-312-S2.PDF]

[10%]

Welcome and thank you for supporting this research project on
neglected and orphan diseases.

This is the second of two rounds of the project's Delphi survey.
The survey will take about 15-20 minutes to complete. The questionnaire can be accessed until August 6, 2008.

Next

Cancel

[20%]

Introduction to the second round

We would like to sincerely thank all participants of the first survey for the time and effort they took to contribute to this research project. We have received many suggestions for additional items and for revising existing items of the questionnaire. The questionnaire for the second round has been expanded and modified accordingly.

We have also received a considerable number of explanatory comments, which we have included as pdf-documents in the questionnaire; you can open the documents by clicking on a link on the corresponding page.

To show the first round's frequency distributions, each page of this modified questionnaire contains tabulations. A click on a blue questionmark on each page opens a pop-up-window to display the graphs.

Before starting the questionnaire, would you like to read participants' comments on the survey from the first round? Please click on the following link to open the pdf-document which contains the comments. comments_on_the_survey.pdf

The questionnaire starts on the following page. Thank you again for contributing your expertise to this survey!

Back Next

Cancel

[30%]

I. Neglected Diseases

Neglected diseases are disease states where there are inadequate, ineffective or no means to prevent, treat, diagnose or cure them. (WHO/CIPIH)

What, in your opinion, are the most important causes for this deficit? Please rate the items below. *The list has been modified and expanded following participants' suggestions in the first round.*

To see the frequency distributions from the first round, please click on the blue questionmark.

Here you find the suggestions made by participants in the first round: causes_for_r_d_deficit.pdf

Technical advice: Please use your left mouse button to click the button of your choice. You may change your answer by clicking another button.

	most important	important	unimportant	least important	no judgement
1) Disease-specific research difficulties (unknown etiology, lack of research material)	○	○	○	○	○
2) Inadequate research priorities in private sector R&D	○	○	○	○	○
3) Lack of awareness /visibility of neglected diseases	○	○	○	○	○
4) Lack of health-needs driven priority setting in public funding	○	○	○	○	○
5) No or inadequate access to effective drugs for neglected diseases	○	○	○	○	○
6) No or inadequate health delivery infrastructure and staff in developing countries	○	○	○	○	○
7) No or inadequate incentives for the private sector to invest into R&D for neglected diseases	○	○	○	○	○
8) No or inadequate private sector investment into R&D for neglected diseases	○	○	○	○	○
9) No or inadequate research coordination	○	○	○	○	○
10) No or inadequate research infrastructure in countries with neglected diseases	○	○	○	○	○

	most important	important	unimportant	least important	no judgement
11) No or ineffective drugs for neglected diseases	○	○	○	○	○

12) No or insufficient direct public funding for research and development (R&D) for neglected diseases	○	○	○	○	○
13) No or insufficient sustainability of public funding for R&D for neglected diseases	○	○	○	○	○
14) Poverty as disease-proliferating factor (i.a. inadequate prevention, inadequate housing, lack of clean water) in endemic countries	○	○	○	○	○
15) Poverty as reason for market failure (perception of no market for drugs, insufficient R&D)	○	○	○	○	○

Would you like to add a comment?
Please fill in the text field.

Back Next

Cancel

[40%]

II. Orphan diseases

In the first round of the survey we asked the participants whether they were familiar with orphan drug laws and, if they were, to judge the laws' performance. Following are the frequency distributions for these questions.

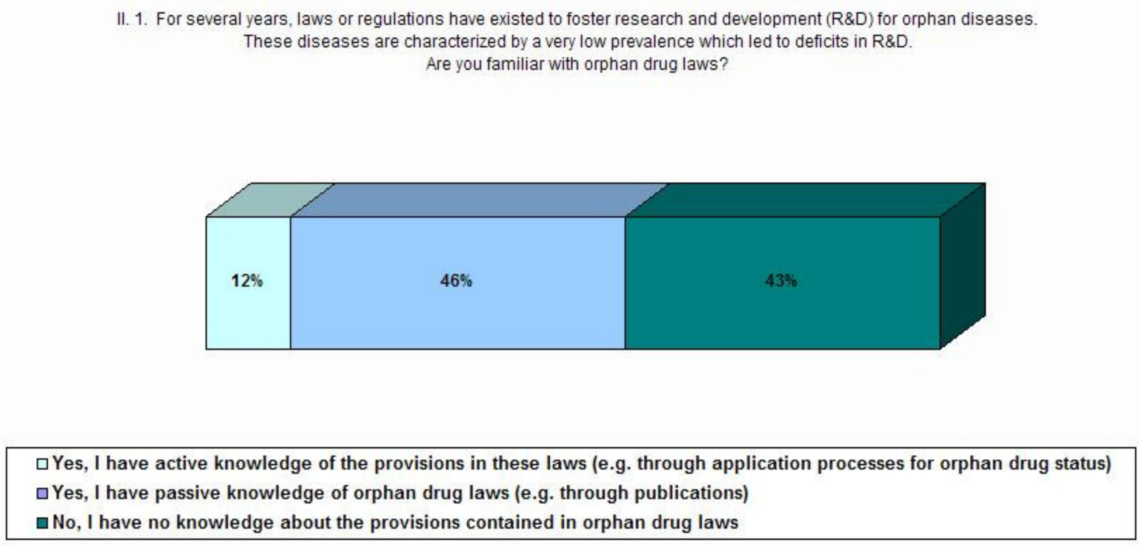

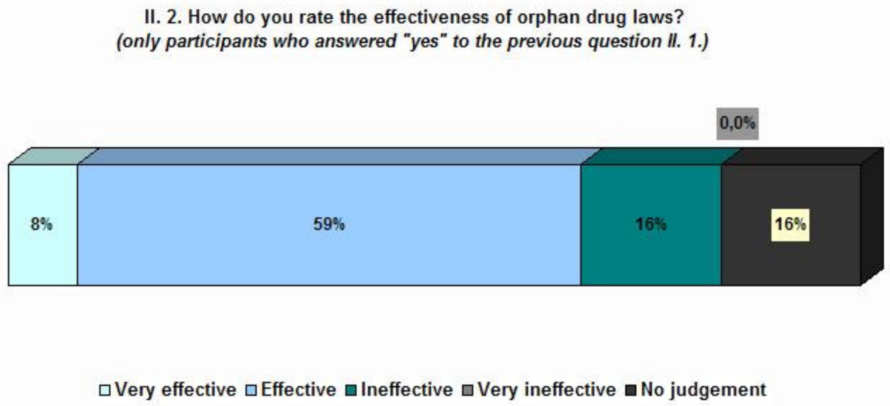

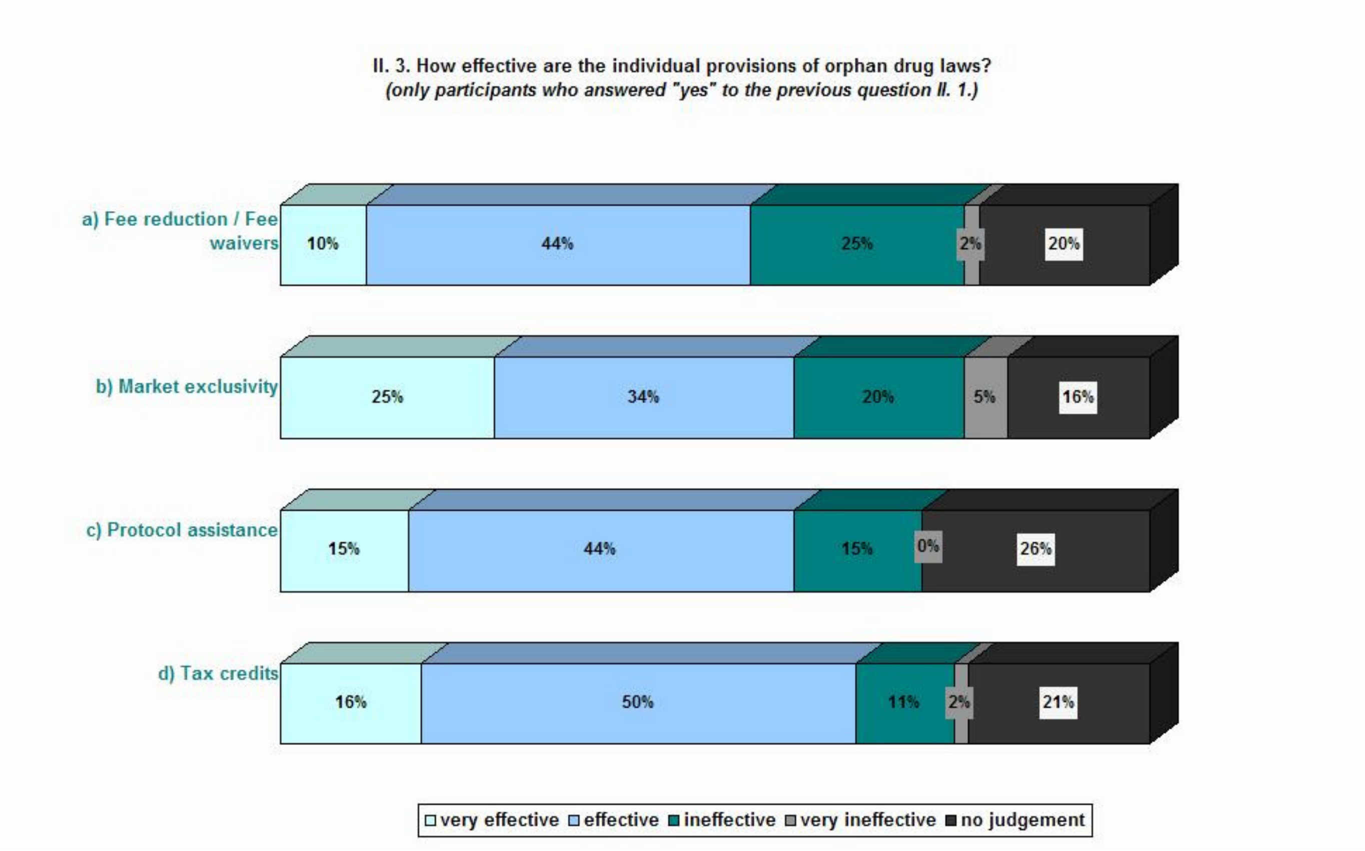

Would you like to comment on these outcomes?
Please fill in the text field.

Back Next

Cancel

[50%]

III. Orphan drug laws contain definitions of what constitutes an orphan disease. In the first round of survey, we asked participants to suggest criteria which could make up a definition of "neglected diseases".

We have subsumed these suggestions into the categories below.

Please indicate how important you consider each item for a definition of neglected diseases.
Here you find the complete list of suggestions:criteria_for_nd_definition.pdf

Please click on the button of your choice. You may de-select your choice by clicking another button.

	most important	important	unimportant	least important	no judgement
1) Absence of effective treatment and lack of ongoing research	○	○	○	○	○
2) Lack of awareness / visibility of relevant diseases	○	○	○	○	○
3) Disease severity: life-threatening, serious, debilitating, chronic	○	○	○	○	○
4) Economic situation of affected population	○	○	○	○	○
5) Lack of access to existing effective treatment	○	○	○	○	○
6) Prevalence, burden of disease	○	○	○	○	○

Would you like to add a comment?
Please fill in the text field.

Back Next

Cancel

[60%]

***Definition "Desirability"**

Very desirable:
extremely beneficial / will have a positive effect and little to no negative effect

Desirable:
beneficial / will have a positive effect and little to no negative effect

Undesirable:
harmful / will have a negative effect

Very undesirable:
extremely harmful / will have a major negative effect

IV. New options for neglected diseases?

Following is a list of measures to promote medical research and development.

a) How desirable* are these measures to foster R&D for neglected diseases? *The list has been modified and expanded following participants' suggestions in the first round.*

To see the frequency distributions from the first round, please click on the blue questionmark.

Here you find the suggestions made by participants in the first round:
measures_to_promote_r_d_for_neglected_diseases.pdf

Technical advice: Please use your left mouse button to click the button of your choice. You may change your answer by clicking another button.

	very desirable	desirable	undesirable	very undesirable	no judgement
1) Abolish patents	○	○	○	○	○
2) Association of biotechnology to health systems for better delivery of goods	○	○	○	○	○
3) Building innovation clusters for low-profit oriented R&D in developing countries	○	○	○	○	○
4) Building research, technical and regulatory capacity in developing countries	○	○	○	○	○
5) Competitive grants to publicly fund research	○	○	○	○	○
6) Contribution by foreign donors towards capacity building and strenthening the research infrastructure in developing countries	○	○	○	○	○
7) Development to phase III trials by public laboratories	○	○	○	○	○

	very desirable	desirable	undesirable	very undesirable	no judgement
8) Educate / inform the public about the individual and societal burden of disease of neglected diseases	○	○	○	○	○
9) Establishment of accountability systems for funds received	○	○	○	○	○
10) Establishment of an international health-needs driven R&D agenda matched to technological opportunities	○	○	○	○	○
	**very desirable**	**desirable**	**undesirable**	**very undesirable**	**no judgement**
11) Establishment of public (or affordable) preclinical research facilities	○	○	○	○	○
12) Exclusive funds for R&D / budgetary set-asides exclusively for purchasing medicinces for neglected diseases	○	○	○	○	○
13) Exemption of drugs from market exclusivity	○	○	○	○	○
14) Existing patent regulations (e.g. WTO-TRIPS, Doha-Declaration)	○	○	○	○	○
15) Fee reduction / Fee waivers (e.g. for marketing approval, scientific advice)	○	○	○	○	○
16) Global funders forum to set priorities	○	○	○	○	○
17) Government support and funds for multilateral efforts (e.g. WHO-TDR)	○	○	○	○	○
18) Incentives for the private sector (e.g. advance market commitments, governmental incentives)	○	○	○	○	○
19) Include neglected diseases in university curricula	○	○	○	○	○
20) Interconnection between research projects on different neglected diseases	○	○	○	○	○
	**very desirable**	**desirable**	**undesirable**	**very undesirable**	**no judgement**
21) Interdisciplinary research cooperation with e.g. veterinary medicine, traditional medicine, epidemiology	○	○	○	○	○

22) International / transcontinental research cooperation involving researchers from developing countries					
23) International regulations regarding the private sector					
24) Link between neglected disease R&D and research and clinical care for priority diseases such as HIV, TB or Malaria					
25) Lower private sector influence on R&D priority setting					
26) Market exclusivity					
27) Neglected disease R&D as priority in relevant European Union funding programs (e.g. FP7)					
28) New alternative juridical instruments which allow governments to foster essential health research and development					
29) Obligation for national governments to invest into neglected disease R&D					
30) Obligation for the private sector to invest x % of profit made from other drugs/treatments into neglected diseases					

	**very desirable**	**desirable**	**undesirable**	**very undesirable**	**no judgement**
31) Open source regulations (e.g. for scientific data or compound / molecule libraries)					
32) Parallel measures to improve access to health care and medicines					
33) Patent pools / more flexible patent laws to improve access to research tools					
34) Price increases (10-20%) for brandname drugs paid by public health programs to invest this profit in neglected disease R&D					
35) Private donations to "real" pharmaceutical companies to develop drugs for neglected					

diseases					
36) Prize funds with prizes awarded based on degree of innovation	○	○	○	○	○
37) Protocol and regulatory advice / assistance to neglected disease R&D projects	○	○	○	○	○
38) Public-private partnerships	○	○	○	○	○
39) Raise awareness among policy makers for the impact of neglected diseases on development	○	○	○	○	○
40) Raising the scientific profile of neglected disease research (better career/publication opportunities)	○	○	○	○	○
	**very desirable**	**desirable**	**undesirable**	**very undesirable**	**no judgement**
41) Reorganize intellectual property rights as intellectual monopoly privileges	○	○	○	○	○
42) Requirement for developing countries to include research with an adequate budget in all health programs	○	○	○	○	○
43) Royalty arrangements for the benefit of neglected disease R&D if private sector receives exclusive license on government-owned invention for any disease	○	○	○	○	○
44) Selective investment (as incentive) in companies which invest in neglected disease R&D	○	○	○	○	○
45) Separation of innovation incentives from drug prices	○	○	○	○	○
46) Sharing or transfer of technology to developing countries	○	○	○	○	○
47) Simplified / fast-track funding procedures	○	○	○	○	○
48) Tax credits / tax incentives	○	○	○	○	○
49) Treaty on cost-effectiveness of new health technologies linked to a competitive tender system	○	○	○	○	○

50) Voucher systems in developed markets (as with the FDA) for other products ○ ○ ○ ○ ○

Would you like to add a comment?
Please fill in the text field.

Back Next

Cancel

[70%]

Definition "Feasibility"

Definitely feasible:
no hindrance to implementation / no political roadblocks / acceptable to the public

Possibly feasible:
some indication this is implementable / minor political roadblocks / further consideration or preparation to be given to public reaction

Possibly unfeasible:
some indication this is unworkable / severe political resistances / difficult to communicate to the public

Definitely unfeasible:
all indications are negative / politically unworkable / cannot be implemented

b) According to your experiences and judgement, how feasible* is it to implement these measures to foster R&D for neglected diseases?

Technical advice: Please use your left mouse button to click the button of your choice. You may change your answer by clicking another button.

	definitely feasible	possibly feasible	possibly unfeasible	definitely unfeasible	no judgement
1) Abolish patents	○	○	○	○	○
2) Association of biotechnology to health systems for better delivery of goods	○	○	○	○	○
3) Building innovation clusters for low-profit oriented R&D in developing countries	○	○	○	○	○
4) Building research, technical and regulatory capacity in developing countries	○	○	○	○	○
5) Competitive grants to publicly fund research	○	○	○	○	○
6) Contribution by foreign donors towards capacity building and strenthening the research infrastructure in developing countries	○	○	○	○	○
7) Development to phase III trials by public laboratories	○	○	○	○	○
8) Educate / inform the public about the individual and societal burden of disease of neglected diseases	○	○	○	○	○

9) Establishment of accountability systems for funds received					
10) Establishment of an international health-needs driven R&D agenda matched to technological opportunities					
	**definitely feasible**	**possibly feasible**	**possibly unfeasible**	**definitely unfeasible**	**no judgement**
11) Establishment of public (or affordable) preclinical research facilities					
12) Exclusive funds for R&D / budgetary set-asides exclusively for purchasing medicinces for neglected diseases					
13) Exemption of drugs from market exclusivity					
14) Existing patent regulations (e.g. WTO-TRIPS, Doha-Declaration)					
15) Fee reduction / Fee waivers (e.g. for marketing approval, scientific advice)					
16) Global funders forum to set priorities					
17) Government support and funds for multilateral efforts (e.g. WHO-TDR)					
18) Incentives for the private sector (e.g. advance market commitments, governmental incentives)					
19) Include neglected diseases in university curricula					
20) Interconnection between research projects on different neglected diseases					
	**definitely feasible**	**possibly feasible**	**possibly unfeasible**	**definitely unfeasible**	**no judgement**
21) Interdisciplinary research cooperation with e.g. veterinary medicine, traditional medicine, epidemiology					
22) International / transcontinental research cooperation involving researchers from developing countries					
23) International regulations regarding the private sector					

24) Link between neglected disease R&D and research and clinical care for priority diseases such as HIV, TB or Malaria	○	○	○	○	○
25) Lower private sector influence on R&D priority setting	○	○	○	○	○
26) Market exclusivity	○	○	○	○	○
27) Neglected disease R&D as priority in relevant European Union funding programs (e.g. FP7)	○	○	○	○	○
28) New alternative juridical instruments which allow governments to foster essential health research and development	○	○	○	○	○
29) Obligation for national governments to invest into neglected disease R&D	○	○	○	○	○
30) Obligation for the private sector to invest x % of profit made from other drugs/treatments into neglected diseases	○	○	○	○	○

	**definitely feasible**	**possibly feasible**	**possibly unfeasible**	**definitely unfeasible**	**no judgement**
31) Open source regulations (e.g. for scientific data or compound / molecule libraries)	○	○	○	○	○
32) Parallel measures to improve access to health care and medicines	○	○	○	○	○
33) Patent pools / more flexible patent laws to improve access to research tools	○	○	○	○	○
34) Price increases (10-20%) for brandname drugs paid by public health programs to invest this profit in neglected disease R&D	○	○	○	○	○
35) Private donations to "real" pharmaceutical companies to develop drugs for neglected diseases	○	○	○	○	○
36) Prize funds with prizes awarded based on degree of innovation	○	○	○	○	○

37) Protocol and regulatory advice / assistance to neglected disease R&D projects	○	○	○	○	○
38) Public-private partnerships	○	○	○	○	○
39) Raise awareness among policy makers for the impact of neglected diseases on development	○	○	○	○	○
40) Raising the scientific profile of neglected disease research (better career/publication opportunities)	○	○	○	○	○

	**definitely feasible**	**possibly feasible**	**possibly unfeasible**	**definitely unfeasible**	**no judgement**
41) Reorganize intellectual property rights as intellectual monopoly privileges	○	○	○	○	○
42) Requirement for developing countries to include research with an adequate budget in all health programs	○	○	○	○	○
43) Royalty arrangements for the benefit of neglected disease R&D if private sector receives exclusive license on government-owned invention for any disease	○	○	○	○	○
44) Selective investment (as incentive) in companies which invest in neglected disease R&D	○	○	○	○	○
45) Separation of innovation incentives from drug prices	○	○	○	○	○
46) Sharing or transfer of technology to developing countries	○	○	○	○	○
47) Simplified / fast-track funding procedures	○	○	○	○	○
48) Tax credits / tax incentives	○	○	○	○	○
49) Treaty on cost-effectiveness of new health technologies linked to a competitive tender system	○	○	○	○	○
50) Voucher systems in developed markets (as with the FDA) for other products	○	○	○	○	○

Would you like to add a comment?
Please fill in the text field.

Back Next

Cancel

[80%]

V. In the first round of this survey, we asked whether participants considered it desirable and feasible to have a regulatory instrument (a law, regulation or treaty) to foster R&D for neglected diseases.
To see the frequency distribution of answers from the first round, please click on the blue questionmark.

Here you find the comments made by survey participants on the desirability of a regulatory instrument: desirability_of_a_regulatory_instrument.pdf and on the feasibility of such an instrument: feasibility_of_a_regulatory_instrument.pdf

a) In the light of the views expressed by the survey participants on this matter, do you consider it desirable to have a regulatory instrument to foster R&D for neglected diseases?

Technical advice: Please use your left mouse button to make your choice. You may de-select your choice by clicking another button.

very desirable	desirable	undesirable	very undesirable	no judgement
○	○	○	○	○

b) In the light of the views expressed by the survey participants on this matter, do you consider it feasible to have a regulatory instrument to foster R&D for neglected diseases?

Technical advice: Please use your left mouse button to make your choice. You may de-select your choice by clicking another button.

definitely feasible	possibly feasible	possibly unfeasible	definitely unfeasible	no judgement
○	○	○	○	○

Would you like to add a comment?
Please fill in the text field.

Back Next

Cancel

[90%]

VI. You have completed the questionnaire. In conclusion, may we ask you again to give us some demographic information. Collecting this information again will allow us to determine whether the composition of the panel has changed between the first and the second round of survey. Thank you for your support!

a) What is your professional background?
(Please click on the blue questionmark to see the frequency distribution from the first round)

Technical advice: Please use your left mouse button to check boxes.
You may check more than one box.

- ☐ Biology / Biomedical Sciences
- ☐ Economy
- ☐ Law
- ☐ Medicine
- ☐ Pharmaceutical Sciences
- ☐ Political Science
- ☐ Public Health
- ☐ Veterinary medicine
- ☐ Other

b) What is your current professional affiliation?
(Please click on the blue questionmark to see the frequency distribution from the first round)

Technical advice: Please use your left mouse button to check boxes. You may change your answer by clicking another button.

- ○ Academia
- ○ National government/parliament
- ○ Industry
- ○ International organization
- ○ Non-governmental organization
- ○ Public Private Partnership
- ○ Other

c) Your place of residence is in a
(Please click on the blue questionmark to see the frequency distribution from the first round)

Technical advice: Please use your left mouse button to check boxes. You may change you answer

by clicking another radio-button.
(A click on the blue questionmark opens the frequency distribution of the 1st round)

- Developed country
- Developing country
- Threshold country/emerging market

Would you like to add a final comment on this survey?
Please fill in the text field.

Back Next

Cancel

[100%]

Thank you for participating in this Delphi survey!

This was the last of two rounds. We will now evaluate the outcome of the survey and send you our first results as soon as possible.

Close window
